# Communication and animal observation in livestock farming – pilot study of a teaching project in veterinary education

**DOI:** 10.3205/zma001457

**Published:** 2021-03-15

**Authors:** Sara Trittmacher, Anne Schnepf, Christin Kleinsorgen, Henrik Detlefsen, Johannes Hessler, Amely Campe, Isabel Hennig-Pauka

**Affiliations:** 1 Stiftung Tierärztliche Hochschule Hannover, Außenstelle für Epidemiologie in Bakum, Bakum, Germany; 2 Stiftung Tierärztliche Hochschule Hannover, Institut für Biometrie, Epidemiologie und Informationsverarbeitung, Hannover, Germany; 3 Stiftung Tierärztliche Hochschule Hannover, WHO Collaborating Centre for Research and Training for Health at the Human-Animal-Environment Interface, Hannover, Germany; 4 Stiftung Tierärztliche Hochschule Hannover, ZELDA - Zentrum für E-Learning, Didaktik und Ausbildungsforschung, Hannover, Germany; 5 Tierarztpraxis Bethen, Cloppenburg, Germany; 6 TPL Tierarztpraxis Lastrup-Löningen, Lastrup, Germany

**Keywords:** communication, stock examination of pigs, animal observation, patient-centered training

## Abstract

**Objective: **Within the scope of a teaching project, students of veterinary medicine are to study animal and environmental observation and how to communicate with the persons responsible for animals on pig farms. They will be prepared to reflect on conversational behavior, identify difficult conversational situations and solve them in a goal-oriented way. In addition to piloting, the the aim of the study is to evaluate the teaching project by the students and the teaching staff.

**Methodology: **Animal observation is trained using a virtual tour of a stock farm based on pictures and videos. The didactic approaches Design Thinking and the creative Walt Disney method are used in order to work on a previously prioritized problem. A typical conflict situation in pig farming is simulated in a role-play. Acquired skills are put into practice during a stock examination on the practice day, where the students communicate their observations. Evaluation is conducted using paper-based questionnaires and feedback interviews.

**Results: **Evaluations of the students are generally positive. The desire to include communication studies in the curriculum was expressed several times. For the theoretical teaching units, a larger group of participants is needed to achieve higher interaction through diversity. The acquired knowledge is reliably applied and utilized on the practice day.

**Conclusion: **The theoretical teaching units extensively prepare the students for the practical stock examination and teach basic skills of communication. Some adjustments to the procedure and focus should be made regarding the practical part. Generally, the conveyed information and methods are considered to be important by the students.

## Zusammenfassung

**Zielsetzung:** Im Rahmen eines Lehrprojektes sollen Studierende der Tiermedizin Tier- und Umgebungsbeobachtung und die Kommunikation darüber mit den für die Tiere verantwortlichen Personen auf schweinehaltenden Betrieben lernen. Sie werden darauf vorbereitet, Gesprächsverhalten zu reflektieren, schwierige Gesprächssituationen zu identifizieren und zielführend zu lösen. Neben Pilotierung ist die Evaluation des Lehrprojektes von Seiten der Studierenden sowie des Lehrpersonals Ziel der Untersuchung.

**Methodik: **Die Tierbeobachtung wird anhand eines virtuellen Bestandsrundganges auf Basis von Bildern und Videos geschult. Dabei werden didaktische Ansätze des Design-Thinking und der kreativen Walt-Disney-Methode genutzt, um eine zuvor priorisierte Problemstellung zu bearbeiten. Im Rollenspiel wird eine in der Schweinehaltung typische Konfliktsituation simuliert. Am Praxistag werden erlernte Fähigkeiten während einer Bestandsuntersuchung umgesetzt, bei der die Studierenden ihre Beobachtungen kommunizieren. Die Evaluation erfolgt mittels papierbasierter Fragebögen sowie Feedbackgesprächen.

**Ergebnisse: **Die Evaluationen der Studierenden sind positiv. Mehrfach wurde der Wunsch, Kommunikationslehre in das Curriculum aufzunehmen, geäußert. Für die theoretischen Lehreinheiten ist eine größere Gruppe nötig, um Interaktion durch Diversität zu erreichen. Das Erlernte wird am Praxistag zuverlässig angewandt und umgesetzt.

**Schlussfolgerung: **Die theoretischen Lehreinheiten bereiten die Studierenden ausführlich auf die Bestandsuntersuchung vor und schulen sie in Grundlagen der Kommunikation. Bei der Durchführung des praktischen Teils bieten sich Anpassungen in Ablauf und Schwerpunktsetzung an. Die Studierenden erachten die vermittelten Informationen und Methoden als wichtig.

## 1. Introduction

Students of veterinary medicine focussing on farm animals are confronted with increasingly difficult working conditions in their future field of activity. Hardly any other economic sector experiences a similar area of conflict between animal protection, environmental protection, consumer protection and the economy as livestock farming in Germany. The present goal of livestock management is to maintain a high state of health and welfare of the animals, especially with regard to preventive consumer protection. Compliance with standards has to be ensured as well as prevention of outbreaks of diseases and early detection of illnesses. The veterinarians’ task is to prevent health problems on farms and to minimize their effects by means of appropriate diagnostics and therapy. With their expertise on diseases, infections, animal welfare and food hygiene, veterinarians can act as a link between contrasting realities of consumers and those working in agriculture. For their role as “mediators between the worlds”, they must be trained accordingly. It is not sufficient to teach only veterinary knowledge during the course of studies; empathy and basic communication skills must also be enhanced. Currently, the curriculum of veterinary medicine in Germany does not include any obligatory courses on communication, although the effectiveness of consultations depends on an appreciative communicative exchange between the person keeping the animals and the veterinarian [6], [16], [18]. Contents are offered in the “hidden curriculum” or in elective courses [17], these being mostly aimed at communication regarding small animal or equine practice. In livestock medicine, there are completely different conflicting goals. A large number of different stakeholders are involved in the area of conflicts on farms (including controlling authorities, slaughterhouses, meat marketing industry, banks, air conditioning, construction and stable technicians, producer groups, end consumers). Another major difference is that observation of a whole herd with a variable number of animals rather than one individual animal has to be taken into account when communicating with the animal keeper. According to research findings, the described course focussing on communication during a stock visit is the first of its kind in Germany for the livestock sector, which must be offered as an educational training.

For practicing veterinarians, the expectations and needs of animal caregivers are not always evident [7]. Furthermore, farmers do not necessarily follow the recommendations of veterinarians [32]. Exchange and communication between all parties is the most important instrument, as decisions have to be made in a context of conflicting objectives [18]. Therefore, from a veterinarian’s point of view, there is a major need to be trained in communication [4], [24]. Even though first “entrustable professional activities” have been defined for the livestock sector [9] and communication has been elaborated, especially in bovine herd management [23], [24], there is still a lack of guidelines and in-house training courses on communicative skills. Overall, compliance of farm personnel is a requirement for maintaining animal health. In addition to knowledge and motivation, empathy for the animals and the “attitude” of the person caring for the animals are success factors [22], [31]. The importance of the attitude and personality of an animal keeper, which both influence the human-animal relationship, has been scientifically proven. The human-animal relationship influences stress levels of the animals [31], [33], frequency and quantity of antibiotic agents administered to the animals [30], compliance with biosafety measures [5] and perception and treatment of individual animals experiencing prolonged pain, suffering and harm [13].

In this newly designed course students perform animal observation together with the farm personnel. They receive an insight into what to pay attention to during communication in order to minimize misunderstandings or conflicts. Experience and information gained during a joint stock assessment can lead to changes in attitudes by making people aware of conditions [31]. The transfer of knowledge about different communication models and strategies, role behavior and discussion structure can sharpen the students‘ awareness of conflicts in communicating with animal owners and lead to short-term and sustainable learning success [1], [10]. An innovative approach from industrial research is Design Thinking, which serves the creative development of problem solutions from a users‘ point of view [3]. For this purpose, the perspective of future customers is taken, a problem is identified, solutions are examined, a solution is prioritized and implemented. By taking on the role of an animal caretaker on their farms, students can grasp a problem better and develop creative solutions. Another creative method used in the course is the Walt Disney method [8]. In this method, the roles of dreamer, realist and critic are allocated to the students and solutions to a previously defined problem are discussed, moderated by the lecturer. The resulting consensus takes the needs and goals of the different actors into account. 

Altogether, the objective of this study is the evaluation of a teaching project including communication training, which was developed specifically for the teaching of communicative skills in the field of animal observation and stock management in livestock medicine. Evaluation is carried out from the perspective of the students as well as the teaching veterinarians. The aim is to further develop and optimize the teaching concept.

## 2. Project description – Method

### 2.1. Positioning of the course 

The course was held six times during the winter semester 2019/2020 during the ninth semester of the undergraduate course on veterinary studies [34]. It was offered to students specializing in “Pig and Poultry” at the University of Veterinary Medicine Hannover, Foundation, Germany. In groups of three, students go through fortnightly learning phases at the Clinic for Swine, Small Ruminants and Forensic Medicine, at the Poultry Clinic, the Outpatient Clinic (outpatient treatment of large animals on farms) and the Field Station for Epidemiology (AfE). The AfE, where the new course took place in the second week, is a diagnostic facility based in a region with a high density of pig farms. In this phase of the studies, the theoretically acquired teaching content of the previous years from different disciplines is applied in a practical environment. This requires the analysis of a concrete situation, its evaluation and the first step in the direction of a required action [3].

The course was held alternately by a single lecturer from a group of four veterinarians, all of whom have teaching experience and who are themselves trained in communication strategies and didactic methods within the framework of personnel development. While all four persons were alternately involved in the theoretical part of the course, the practical part was conducted by one of two teachers.

#### 2.2. Theoretical part of the course

The theoretical part of the course is divided into two sections, each with four teaching units. The teaching concepts including time schedule and methods are summarized in table 1 (Tab. 1) and table 2 (Tab. 2).

The first part, which is carried out by the AfE, focuses on the perception of animal signals and environment. In an initiating phase, students perform an analysis of the strengths, weaknesses, opportunities and threats (SWOT analysis [15]) of the AfE, based on their experiences and lessons learned during their first week. In doing so, they become familiar with the method which will be used on the farm as well later on. Within three minutes, at least three ideas are written in each of the quadrants (strengths, weaknesses, opportunities and threats) before the piece of paper is passed on to the next person and the process starts again. Ideas collected this way are then discussed and summarized. An introductory presentation is given about areas in farm animal medicine for which the importance of attitude and personality of the animal owner have been scientifically proven. The first work phase includes the approach of Design Thinking. After the method has been presented to the students, they put themselves in the position of the animal caretaker [3]. Based on this approach, the students experience a virtual tour of a stock farm through the eyes of a farmer. Animal signals and environmental factors are observed and prioritized. The group agrees on one resulting problem. A solution which is acceptable to all participants is worked out in accordance with the Walt Disney method [8]. Afterwards, the planned approach for the practical day on the farm is explained, including key questions to the farmer (see table 3 (Tab. 3)) and individual examination protocols. Finally, remaining questions are answered and results are secured.

The second theoretical section is carried out by the Institute of Biometry, Epidemiology and Information Processing (IBEI).

During the initiation phase, the students are prepared for the following teaching unit with questions regarding their previous experience and knowledge. In the following role-play, a conflict situation that frequently occurs in veterinary herd management of pig farms is simulated. The communication and emotions that arise while roleplaying serve as a basis for following different theoretical approaches. The drama triangle [28], established in transactional analysis and non-violent communication in accordance with Rosenberg [25], serving as a solution strategy, are presented in the first work phase. Subsequently, by classifying a situation from the role-play and formulating a sentence as an exit strategy in accordance with Rosenberg, the theoretically gained knowledge is applied and deepened in practice.

During the second phase of the program, the students first work out needs and tasks of a veterinarian, then the client- contractor model (also known as the principle- agent theory [21]) is explained. In this context, special attention is drawn to different contract models in veterinary medicine, and the special role of the students during the stock examination is elaborated. As the willingness of the farmer to cooperate and change is an essential part of veterinary treatment, the “Rubicon Model of Action Phases” [12] is used to explain how to recognize in which phase of a decision-making process the farmer is. Possible ways of influencing the outcome of the decision-making process are identified.

During the closing phase, the lecturer briefly summarizes the topics covered and clarifies any remaining questions.

#### 2.3. Practical part: Animal observation and communication on the farm

The practical part of the course on a pig farm took place in close cooperation with the veterinarians attending the herd two days after the theoretical part. This follows a fixed schedule (see table 3 (Tab. 3)) so that students can focus on animal observation and communication during the complex stock situation. Six teaching units (4.5 hours) are planned, which vary depending upon the size of the farm and the extent of the problems. While the students look at the environment and animals alone and write down their observations, the teacher conducts an anamnesis interview. In the manner of a “midwife conversation” to survey concerns [27], the most important concern of the farmer is identified by means of open questions and a listening conversation method. This is followed by key questions, which can be meaningful for personality and attitude traits (see table 4 (Tab. 4)). Afterwards, all participants examine the entire animal stock. The students communicate their previously made observations to the farmer in a non-judgmental manner while taking notes on further observations. The focus is on the most urgent problem identified in the anamnesis interview. Animals showing clinical signs related to the identified problem are counted and examined by the students. This enables a quantitative and also a qualitative assessment of the problem, which is then discussed with the farmer. As a behavioral test, a Forced Human Approach Test is performed to assess the human-animal relationship [29]. The examiner defines a specific animal and makes contacts with it with increasing intensity. The possible intensity of contact before escape behavior is shown by the animal determines the score on a scale of one to four. Last of all, a SWOT analysis of the visited farm is performed and discussed with all parties involved.

#### 2.4. Follow-up phase, evaluation and outlook

Following the practical part of the course, students have the opportunity to evaluate each part of the course using an anonymous, paper-based questionnaire and to have an open feedback session. For the evaluation, the teaching staff, the teaching material and the motivation of the students, as well as how they have benefitted from the course are assessed using a five-stage Likert style scale [19] (strongly agree, agree, neutral, disagree, strongly disagree). The evaluations are then descriptively analyzed in form of graphs using SAS 9.4 (SAS Institute Inc., Cary, NC, United States).

By the end of the week, students enter the results of answered key questions into a spreadsheet for later analysis and get time to clarify remaining questions. The teachers evaluate the course based on their own experiences and observations during the different units, the evaluation by the students and the final discussion. All insights gained by evaluation are adopted in future courses. All participating farms will be visited and examined again by different students in the following semester. Until the follow-up visit, the strategic approach as well as the questionnaires are modified according to previously made experiences. The previously defined main problem of the farm is then re-evaluated. 

## 3. Results

### 3.1. Evaluation by the teachers

#### 3.1.1. Theoretical teaching units

The interactive teaching units are well received by the students who participitate with high interest. Presented and developed methods are understood and internalized, which is shown by successful utilization of learned skills during the practical part of the course. The method of SWOT analysis has proven itself in the theoretical and practical part. Through targeted reflection and triggered associations during brainstorming, an analysis of conditions and situations can be quickly carried out, which provides an excellent basis for discussion. 

##### 3.1.2. Practical teaching unit

The main problems, which were worked on during the stock examination are summarized in table 5 (Tab. 5). As practiced theoretically, students are able to take on the perspective of the farmer. Estimations of the livestock’s health score by the students correspond in five of six times to the estimation made by their caregiver.

#### 3.2. Evaluation by the students

**Figure 1:**
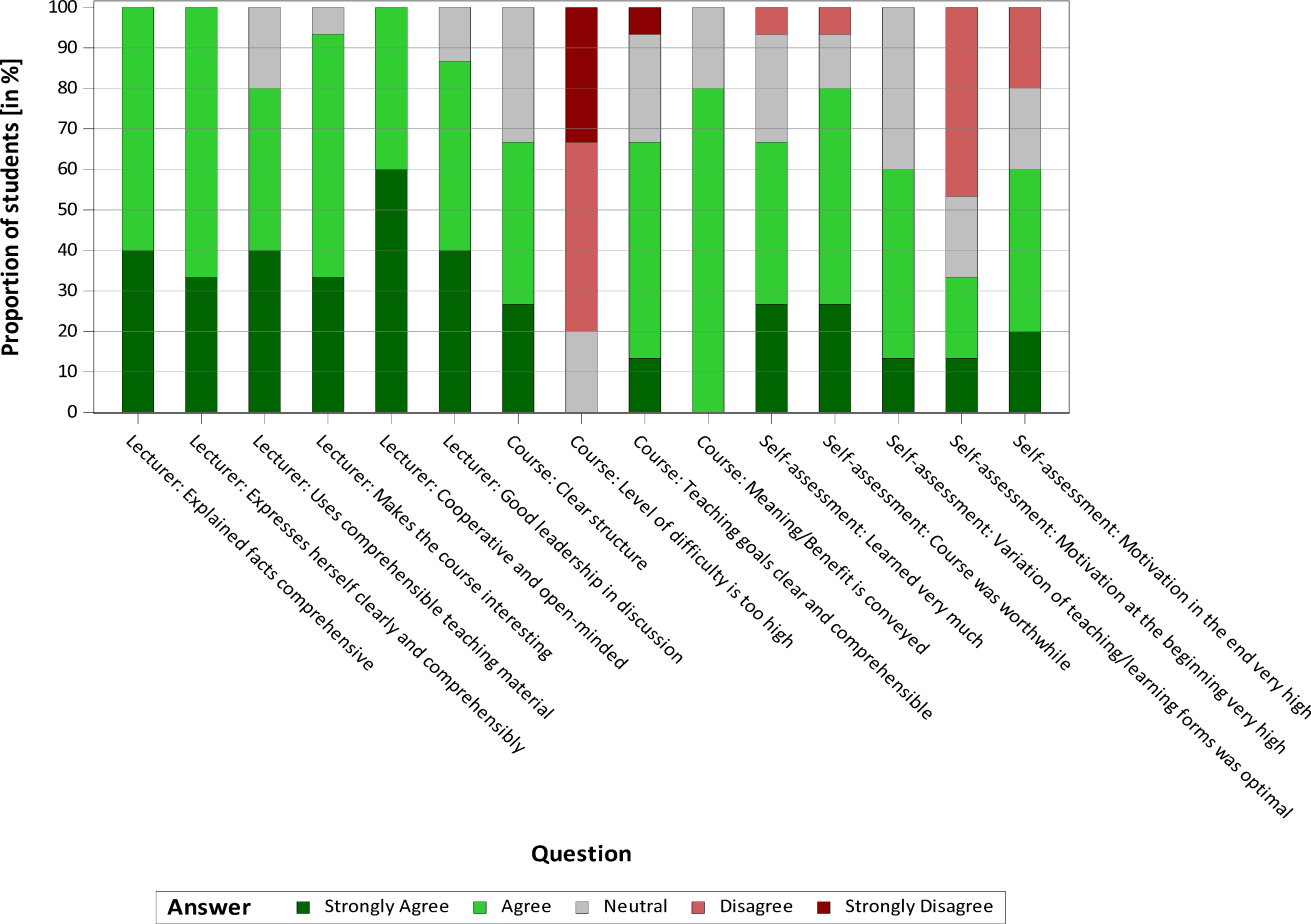
Evaluation of the course "Animal observation in pig farms" by the students (n=15)

**Figure 2:**
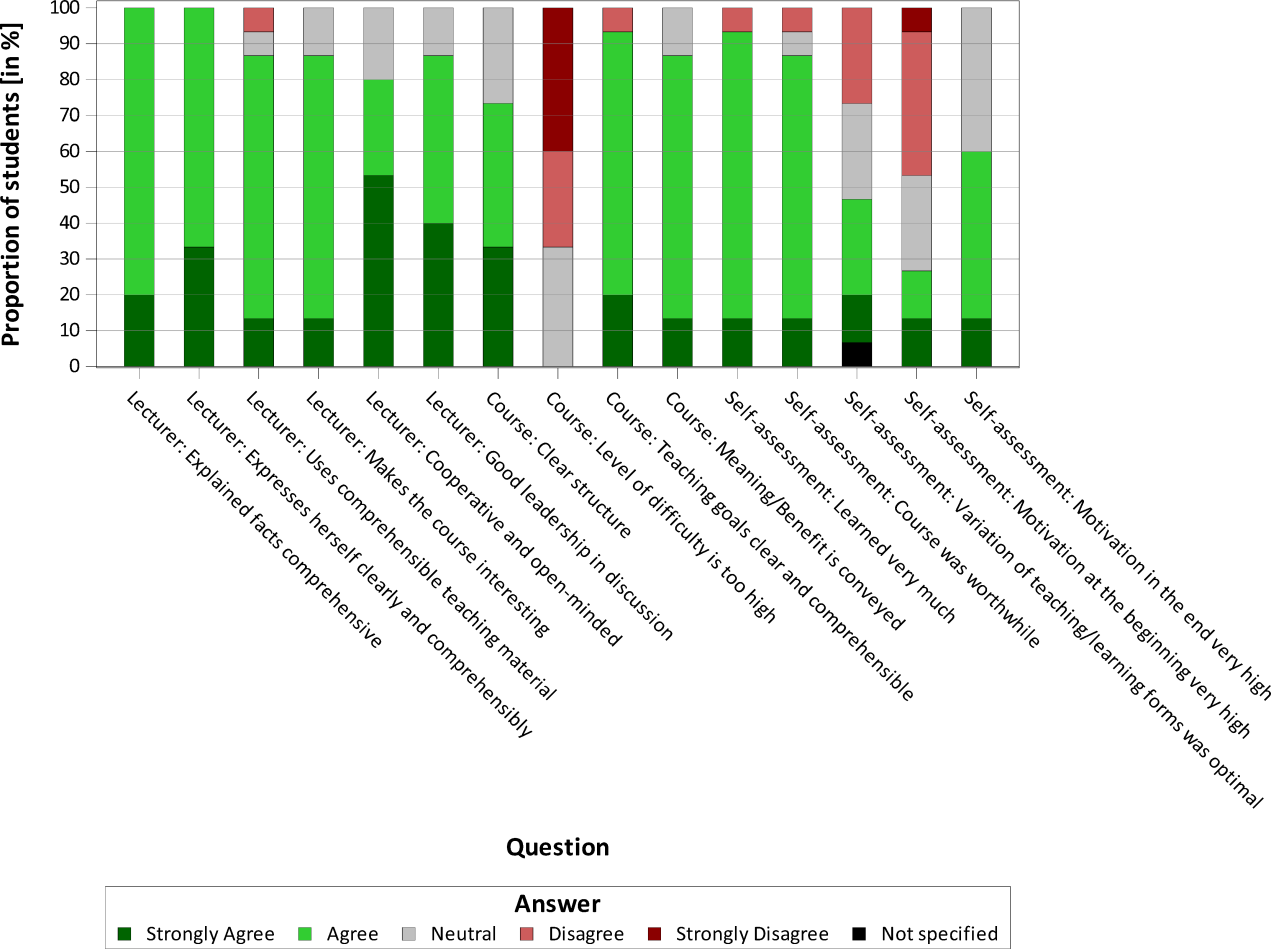
Evaluation of the course "Conflicts between veterinarian and animal owner, which become apparent in communication" by the students (n=15)

**Figure 3:**
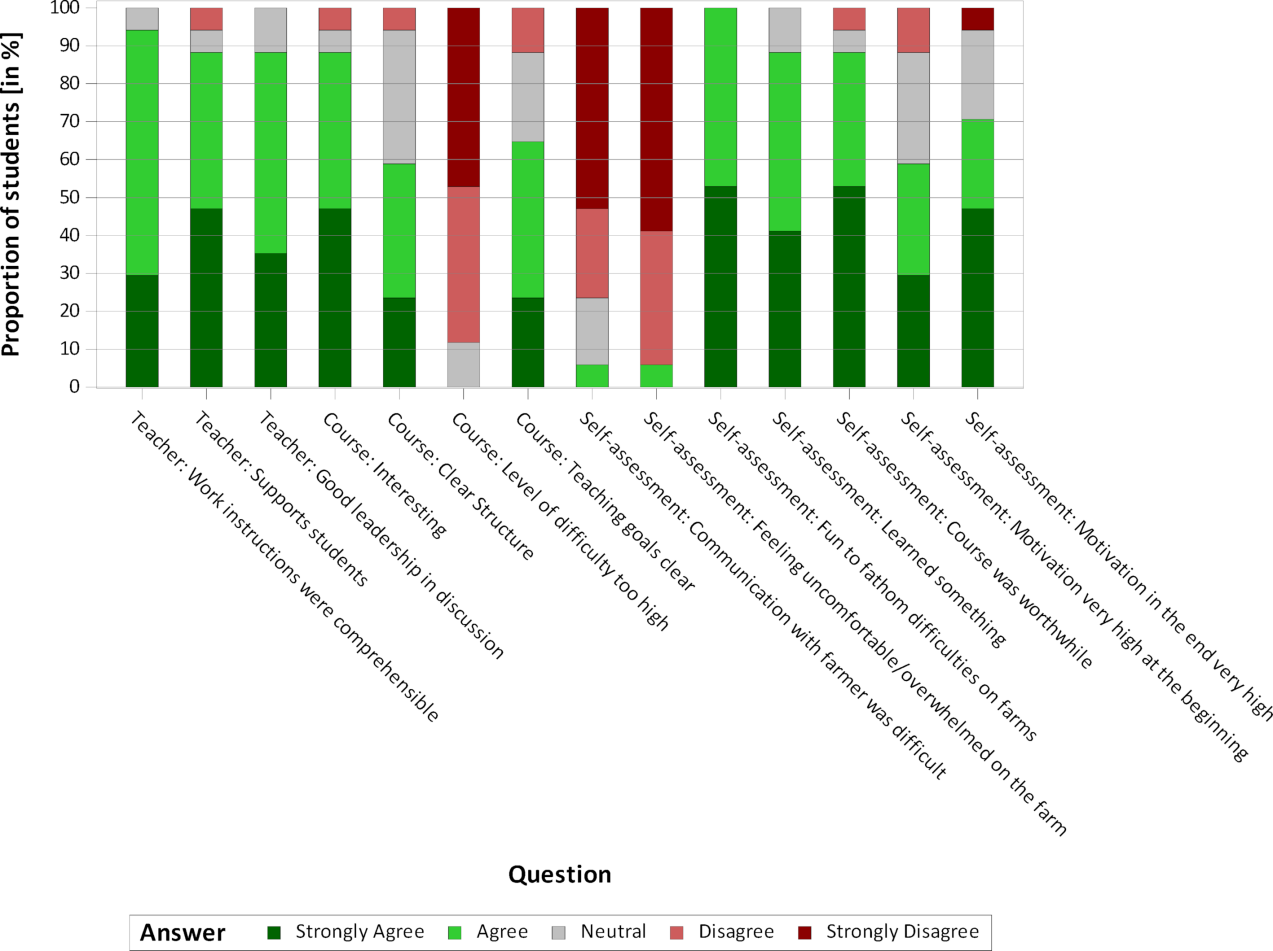
Evaluation of the course "Stock examination and communication" by the students (n=17)

Seventeen participating students stated that they had no special previous knowledge of communication strategies. At the same time, there was an awareness of problems in communication, as the students were able to report on numerous individual situations regarding their encounters with veterinarians and animal owners that had been problematic. Twelve of fifteen students rated the theoretical teaching units as significant and useful in terms of content. At least ten of them stated that they had gained knowledge from the various units. This is reflected in the evaluations of the practical day. Sixteen of seventeen students denied that they felt uncomfortable or overwhelmed during the stock examination. Communicating with the farmer was rated as “rather easy” by fourteen of seventeen students. This shows that the students are prepared at an appropriate level for the practical day by the theoretical teaching units. The degree of difficulty was overall rated as “rather low” by the students. The motivation of the students varied initially but it was slightly improved by the course. Overall, the course was very well received and positively evaluated by the students (see figure 1 (Fig. 1), figure 2 (Fig. 2) and figure 3 (Fig. 3)). In personal discussions with the students, a wish to include teaching units on communication skills in the curriculum was expressed several times. In addition, the necessity to establish this as an essential part of the veterinary education curriculum for all students was expressed.

#### 3.3. Data evaluation

By using statistical evaluation of examination results after follow-up visits, it can be determined whether the developed and suggested solution proposals were effective after implementation. At best, evaluation results then allow conclusions to be drawn about the effectiveness of the veterinary advice developed and provided by the course.

## 4. Discussion

### 4.1. Theory

In order to impart the basics of stock examination to the students, the virtual farm examination has proven itself in the theoretical unit. “Communication in livestock medicine” as a topic was gratefully accepted by students as well as by the veterinarians attending the herds, as they see a great need for it. The student-activating role-play has also proven itself. Through teaching with a focus on theoretical backgrounds, the students gain a deep insight into the respective theory without getting overwhelmed by details at the same time. This teaching approach should be maintained, since frontal teaching is unlikely to achieve the desired learning goals. Especially for the role-play on the topic of communicating with the farmer, each role should be assigned an observing person. Focusing on observation, the additional person perceives verbal and non-verbal communication of their character and describes it in the following discussion. The interactive nature of both theoretical learning units generally requires a larger number of students. For role-plays and SWOT analysis, the number of two to three students is too small. In future courses, the aim is to increase the size of the group to nine students, as the different work phases are enriched by the respective experiences and emotions of several participants.

#### 4.2. Practice

The evaluations of the students suggest that the teacher successfully deals with uncertainties at the farm visit, which enables the students to apply learned strategies in a protected environment and show their learning success (Difficulty of the conversational situation – see figure 3 (Fig. 3)). Following the didactic model of “Constructive Alignment” [2], the learning objectives mentioned above were defined, communicated with the students and teaching methods adapted to them were applied. A systematic learning success control has not yet been developed, but is to be implemented in future courses. For this purpose, a communication checklist based on the Calgary-Cambridge Guide [1], [18] and the Red Interaction Analysis System [26] will be developed, with special emphasis on items specific to stock examinations. In order to be able to give standardized and individual feedback to each student, checklists will be filled out individually for each of them during the course.

Examination time for perceiving the animals and their environment on their own was always fully utilised by all students and usually had to be ended by the teacher. In order to be able to use the learned methods of animal and environment observation, more examination time for their free observation is to be granted to the students. It is planned to have the structured observation form filled out individually in a second phase. In a third phase, the students answers will be compared, commented on by the teacher, enabling the group to objectify their observations.

With one exception, the students evaluated the animal health on each farm in the same way as the person caring for the animals. In herd 1, the farmer evaluated the animal health a little worse. On this farm, the students positively noticed a good structure and cleanliness of the pens as well as good general condition of the animals. This was rated higher than coughing and sneezing of the animals. The symptomatology of the animals did not lead to a disturbed general condition and was predominantly assessed as a problem of the upper respiratory tract. The animal caregiver, on the other hand, suffered from the fact that antibiotics had to be used more often during the rearing phase, whenever coughing became more frequent. Therefor his primary concern was to reduce the clinical symptoms. This discrepancy clarifies that the situation the students see on the farms is always a snapshot, so expectations for future developments on the farms should not be too high. First and foremost, this is a teaching course. Improvements in stock health is in the hands of the animal caregiver and the stock-attending veterinarian.

During the joint stock examination, guiding key questions, which draw attention to the important things, should be preferably asked, e.g., “How is the lying behavior of the animals before they are startled by my appearance”. The teacher's protocol must also be revised. For example, the answer to the question about sick animals on the farm (see table 4 (Tab. 4), question 9) cannot be meaningfully evaluated. Sick animals occur in so many places on farms and in so many degrees of severity that the term “sick” itself is interpreted in many ways by different recipients. It seems more reasonable to look at the equipment and occupancy of the sick pens and to count individual animals that would have to be separated from a veterinary point of view but have not yet been separated. Whether the fact that such animals exist on a farm speaks for a contradiction in self-awareness and awareness of others or for bad animal observation by the caretaker can be determined by further questions in the presence of the animal concerned. Even though popular among the students, the Forced Human Approach Test for assessing the human-animal relationship seems redundant, since a holistic evaluation of the results is not possible. For example, fattening pigs can show a negative human-animal relationship because they are so healthy, people rarely have to enter their pen so they are not used to human contact [20]. This test should therefore only be performed if it makes sense from a professional point of view. For example, testing sows in reproduction, where a good human-animal relationship has proven to have a positive effect on fertility [14]. The SWOT analysis is well suited for a concluding, reflective discussion on the farm premises with all parties involved. It reduced complexity, which was shown to be especially important for the students after the confusing situation in the stable. A disadvantage is the superficial analysis given by the method, as there is no prioritization of the collected information and further necessary expert knowledge is missing (e.g., from an economic consultancy firm or a stable construction company). In future, the concluding consolidation phase should also include reflection on the conversations on the farm. For this purpose, the teacher must take on the role of an observer, not only of the animals, but also of the involved people. The most common motivation of the farmer to participate in the event is for animal health to be improved by the provision of professional and free advice. The teacher must serve these wishes, which makes the teaching course demanding. Minor restructuring can create room for both, the teaching veterinarian dedicating the time during which the students pursue their animal observation exclusively to the professional questions of the animal caregiver. 

All in all, the practical teaching unit is extremely demanding for the teacher, as he or she must simultaneously instruct the students, listen to the farmer and observe the animals. Despite planning, unpredictable situations occur, which the teacher has to be able to react to, spontaneously and flexibly. The time required is very high for every person involved. Close supervision and practical references lead to a high amount of information, which gives all participants the feeling the days were eventful and full. In future, the mentoring ratio could be increased to four or five students in terms of effectiveness, without creating additional burdens for teaching staff. Nevertheless, the aspect of “hands-on training” [11] required in veterinary education, especially by the European Association of Establishments for Veterinary Education of Europe (EAEVE), would be guaranteed.

In future, an (economic) benefit gained from the course should be recognizable for the farmer. Whether and how this can be done requires further strategic considerations.

After at least half a year had passed since the joint herd examination, a debriefing of the teaching veterinarians with the veterinarians attending the herds took place. In fact, an improvement in the problems, which had been the mainfocus of the respective course, was observed on all farms. According to the authors, these improvements are not due to seasonal fluctuations or chance. They could be linked by the veterinarians to individual measures, that were initiated after the joint herd examination had been carried out. Whether or not the joint experience on the farm provided the impetus for some of the changes is impossible to answer. However, the overall result is very motivating to continue and to establish the course firmly in the curriculum.

## 5. Conclusion

The theoretically conveyed content extensively prepares the students for the herd examination. In the practical part, the students communicate their observations of the animals and their environment reliably with the persons responsible for the animals. The aim of their conversation is to identify needs, state of mind and conflicting goals in order to find constructive solutions together. The best-case scenario is initiation of improvement processes on the farm, which can be traced back to the effects of the course (joint, empathic animal observation and communication experience of the farmer). Overall, the course is positively evaluated by students and lecturers and is considered necessary.

## Ethics

The project was submitted to the Data Protection Officer of the University of Veterinary Medicine Hannover for examination and approval. The anonymity of the data was guaranteed at all times during the study.

## Funding

Project 123 was financed by the Ministry of Science and Culture of Lower Saxony, Germany within the framework of a call for projects: Innovative teaching and learning concepts: Innovation Plus (2019/20).

## Profiles

**Place of location: **University of Veterinary Medicine Hannover, Foundation, Field Station for Epidemiology, Büscheler Straße 9, 49456 Bakum and Institute for Biometry, Epidemiology and Information Processing and Centre for E-Learning, Didactics and Educational Research, Bünteweg 2, 30559 Hannover.

**Subject of study/professional category: **Veterinary Medicine

**Number of students per year or semester: **200

**Is a longitudinal communication curriculum implemented? **No

**In which semesters are communicative and social skills taught? **1, 2, 9 (mandatory), 1-4, 5-8 (optional electives), 1-11 (Skills Lab, E-Learning).

**Which teaching formats are used? **Mandatory: Lectures, small group exercises incl. conversation simulations with actors and feedback.

Optional: seminars, small group exercises incl. conversation simulations with actors and feedback, e-learning 

**In which semesters are communicative and social competencies examined (formative or relevant to pass and/or graded)? **Formative as feedback interviews

Relevant to pass the practical year (9^th^ semester) in the Department of Small Animal Medicine and Surgery.

**Which examination formats are used? **Feedback interviews, objective structured clinical examination (OSCE)

**Who (e.g. clinic, institution) is in charge of development and implementation?** Centre for E-Learning, Didactics and Training Research

## Current professional roles of the authors

- Sara Trittmacher: Veterinarian, working as Research Associate and Doctoral Candidate at the Field Station for Epidemiology in Bakum, involved in microbiological, molecular biological and serological diagnostics of pig diseases and educating students in practical laboratory activities, project- related development and implementation of the new teaching format.
- Anne Schnepf: Veterinarian, working as Research Assistant and Doctoral Candidate at the Institute of Biometry, Epidemiology and Information Processing, statistical design of experiments and evaluation of data, development and evaluation of personality questionnaires for the veterinary field. Evaluation of studies on the attitude of animal owners, project-related development and implementation of the new teaching format with focus on communication.
- Christin Kleinsorgen: Veterinarian, Research Associate at the E-learning Consulting Department of the Center for E-Learning, Didactics and Educational Research, consulting and training in the use of digital media and the development of teaching and learning programs on the topic of communication and key competencies in veterinary medicine, creating teaching programs, responsible for their integration into the curriculum, courses on the topic of communication.
- Henrik Detlefsen: Veterinarian in specialized pig practice, caring for pig farms with different production types and sizes, focusing on veterinary advice, diagnostics and prevention of diseases, therapy, health management from breeding to fattening, supporting the practical training of students at the University of Veterinary Medicine Hannover, Foundation, specializing in "Pig and Poultry".
- Johannes Hessler: Veterinarian in specialized pig practice, caring for pig farms with different production types and sizes, focusing on veterinary advice, diagnostics and prevention of diseases, therapy, health management from breeding to fattening, supporting the practical training of students at the University of Veterinary Medicine Hannover, Foundation, specializing in "Pig and Poultry".
- Amely Campe: Veterinarian, Research Associate at the Institute of Biometry, Epidemiology and Information Processing, Head of the Working Group "Animal Health", working areas are animal welfare, animal diseases and animal behavior, research on factor diseases and epidemiological analysis methods for complex systems, quantitative and qualitative studies in different animal populations, implementation of new teaching formats with focus on communication in the context of Veterinary Public Health.
- Isabel Hennig-Pauka: Veterinarian, Head of the Field Station for Epidemiology in Bakum, theoretical and practical courses in pig medicine focussing on herd diagnostics, infectious diseases, management and prevention strategies, animal observation. Quality management in the accredited diagnostic laboratory of the institute, research projects in the field of diagnostics and infectious medicine.

## Competing interests

The authors declare that they have no competing interests. 

## Figures and Tables

**Table 1:**
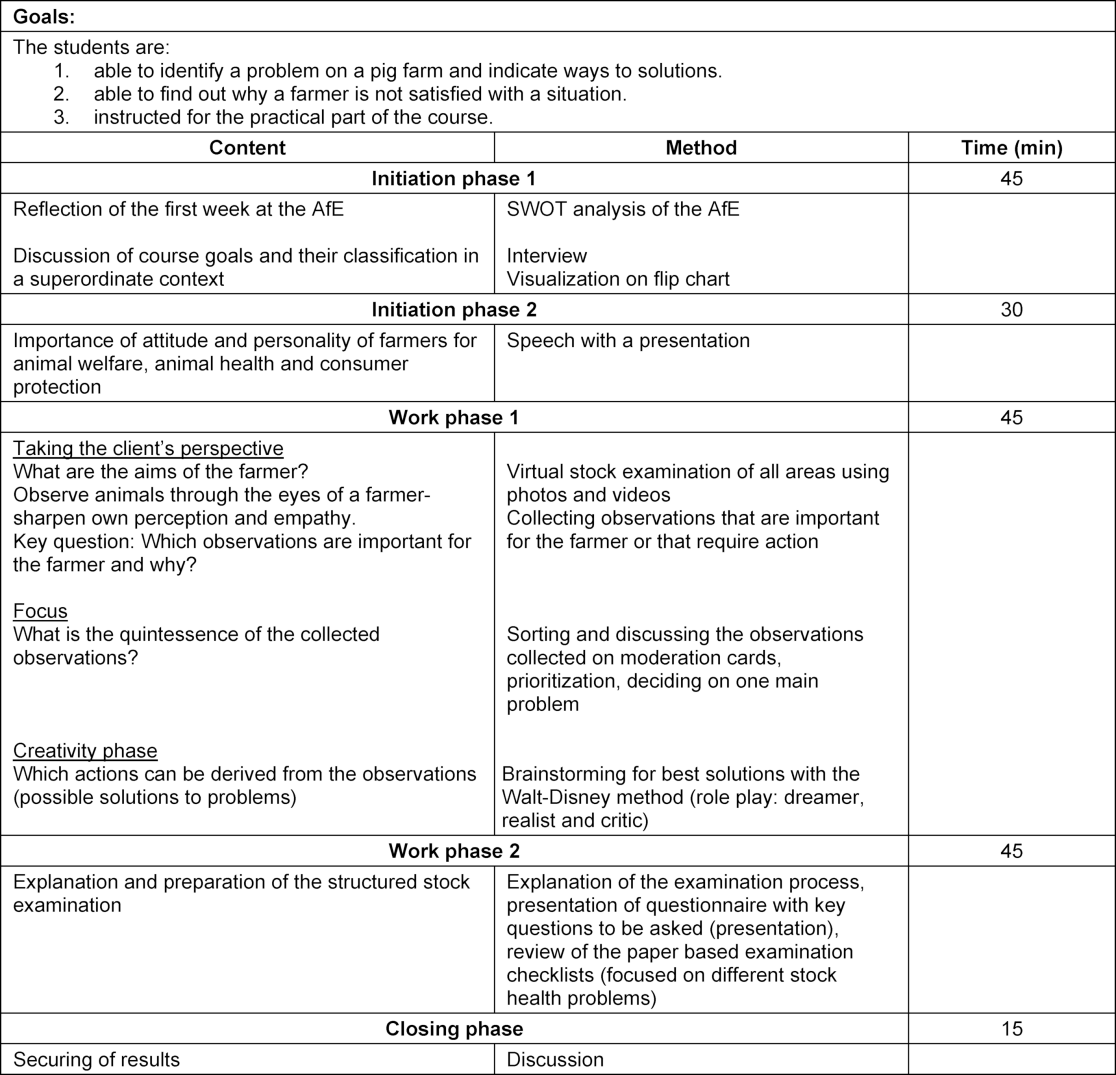
Teaching concept for animal observation and preparation for stock examination

**Table 2:**
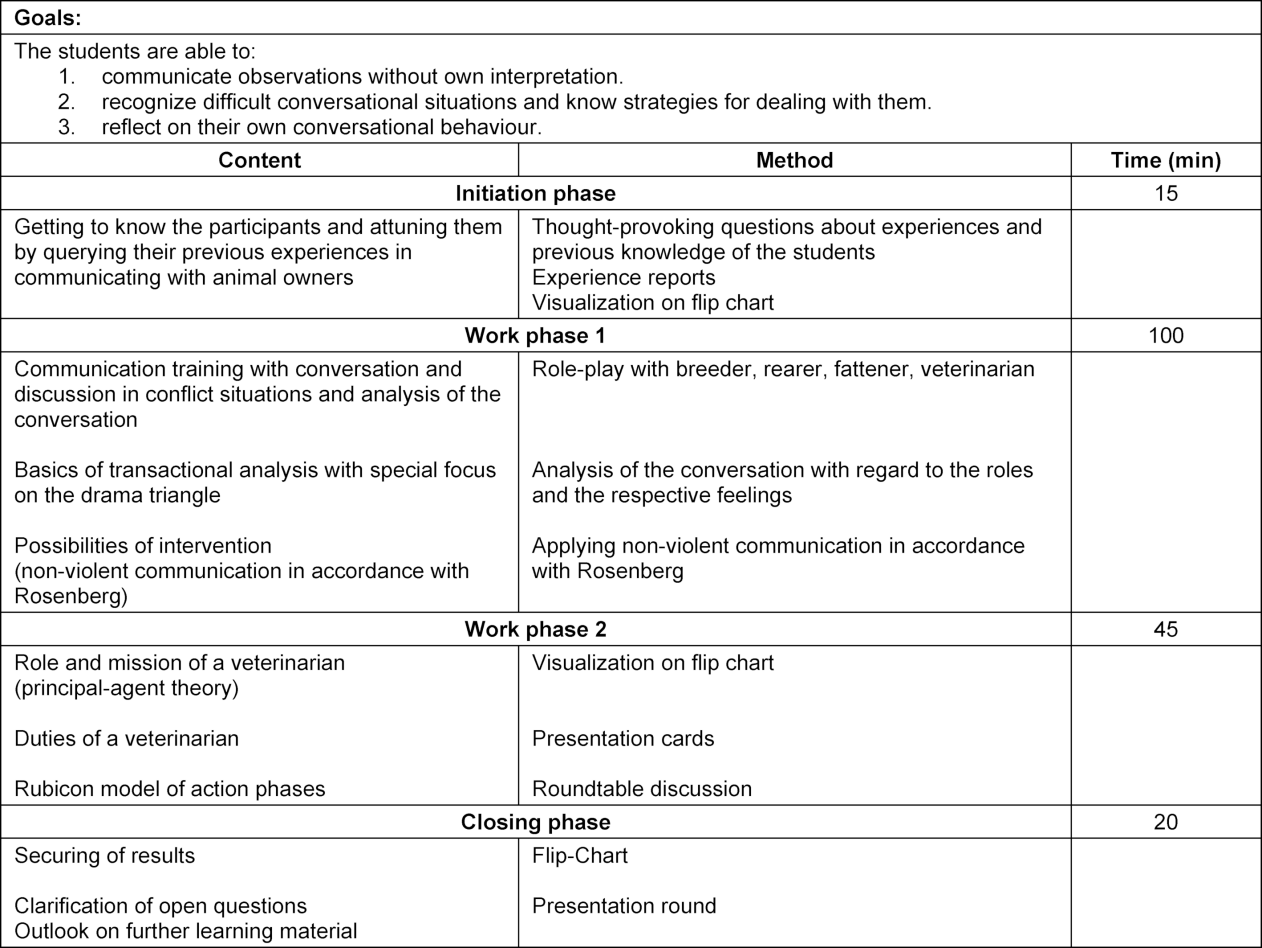
Teaching concept introducing how to communicate with a farmer

**Table 3:**
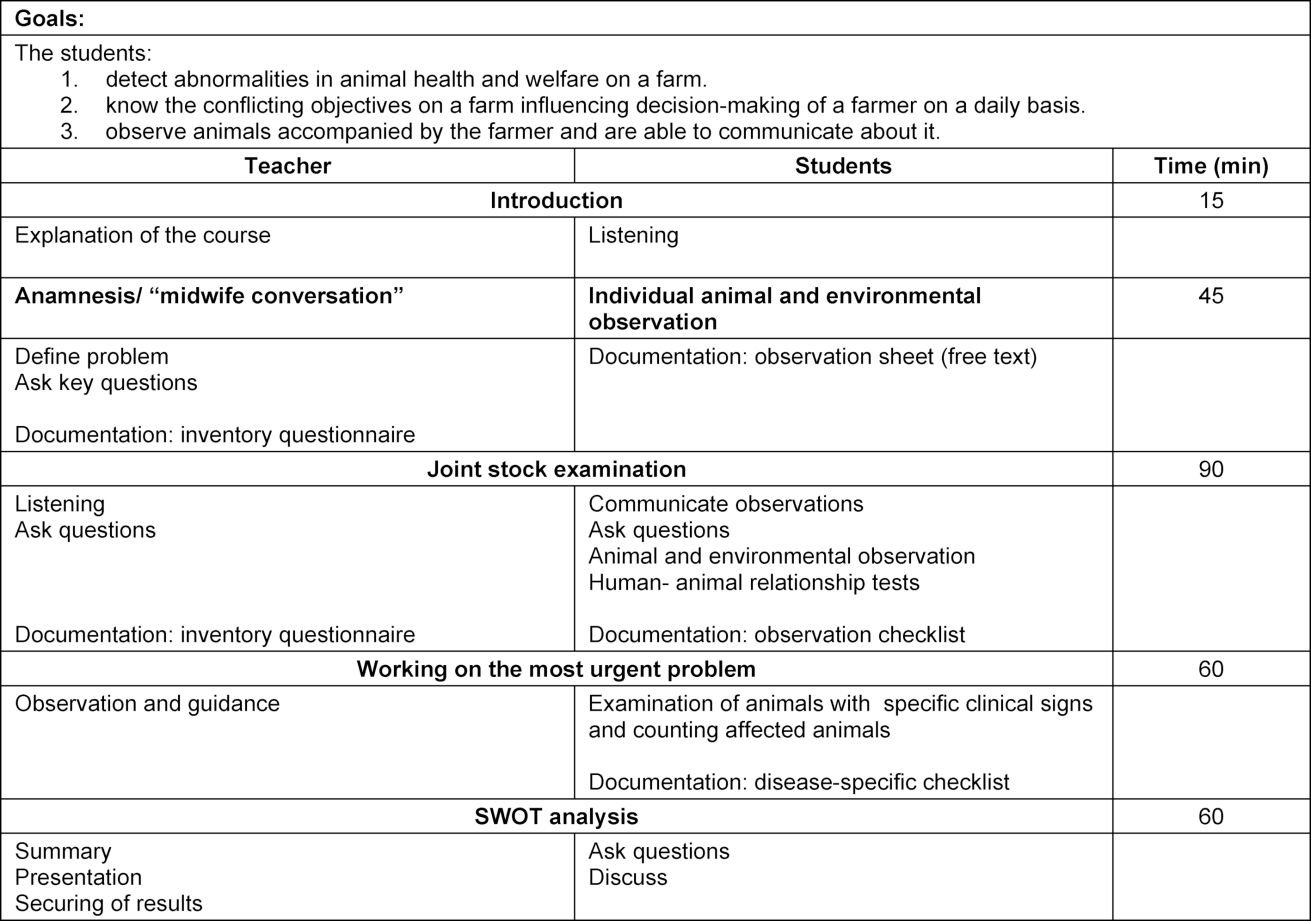
Training concept for animal observation and communication on a farm

**Table 4:**
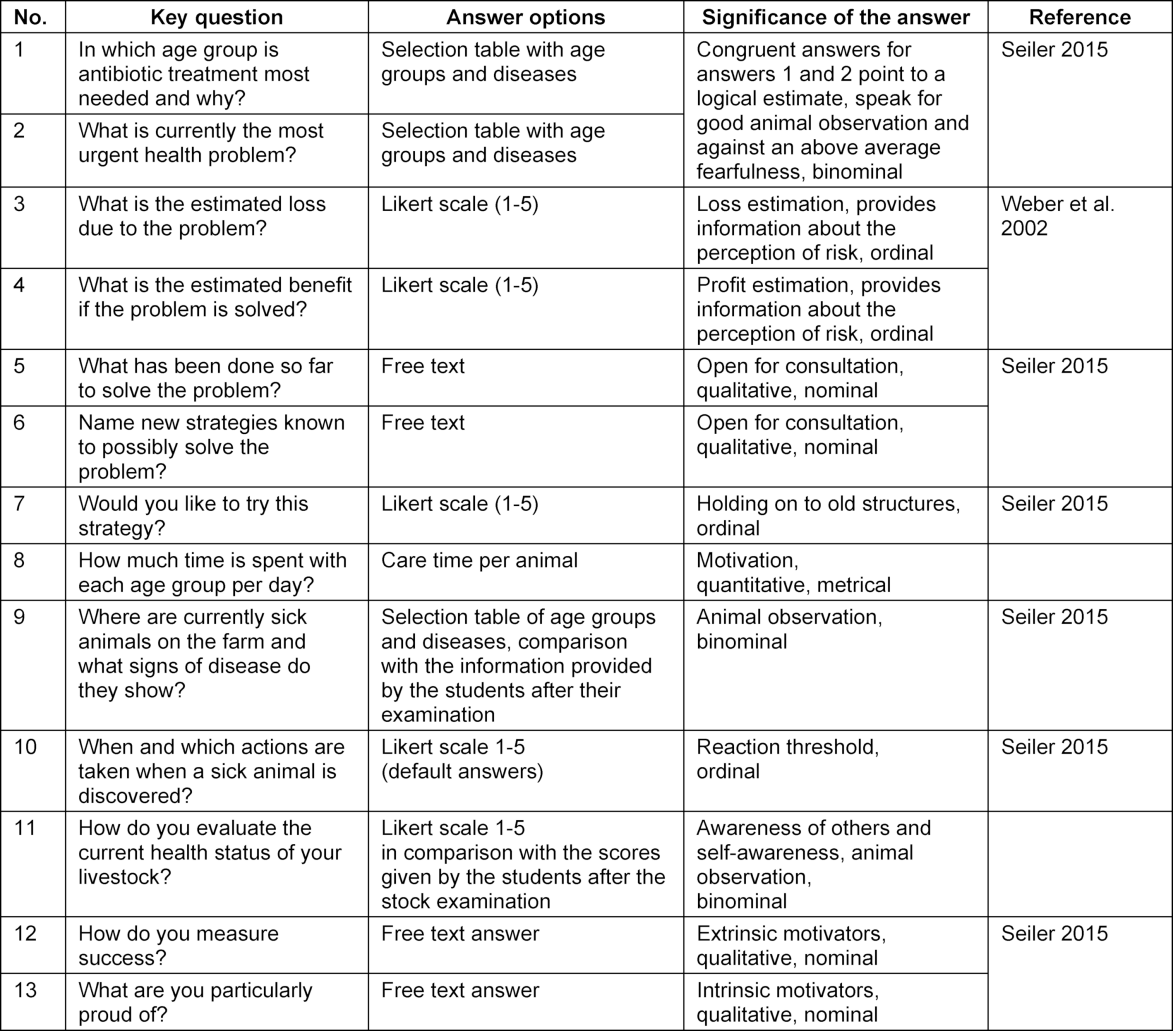
Key questions to the farmer during a stock examination

**Table 5:**
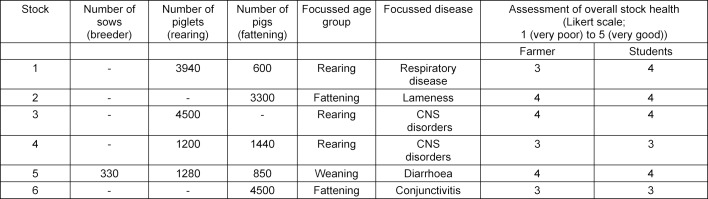
Main topics on the examined pig farms
